# Identification and genotyping of a new subtype of bovine viral diarrhea virus 1 isolated from cattle with diarrhea

**DOI:** 10.1007/s00705-021-04990-7

**Published:** 2021-02-13

**Authors:** Bin Tian, Dongjie Cai, Weiqiang Li, Qinglong Bu, Mingshu Wang, Gang Ye, Jianzhu Liu, Ya Wang, Liping Gou, Jun Yi, Zhicai Zuo

**Affiliations:** 1 Institute of Preventive Veterinary Medicine, Sichuan Agricultural University, Chengdu, 611130 China; 2 Department of Veterinary Clinical Sciences, College of Veterinary Medicine, Sichuan Agricultural University, Chengdu, 611130 China; 3 Ministry of agriculture “Veterinary medicine and veterinary technology” Sichuan scientific experimental observation station, Sichuan Agricultural University, Chengdu, 611130 China; 4 College of Veterinary Medicine, Shandong Agricultural University, Tai’an, 271018 China; 5 Sichuan Academy of Animal Science, Chengdu, 610066 China

## Abstract

In 2019, diarrhea cases occurred on cattle farms in Qionglai and Guang'an, Sichuan Province. Two out of 20 (10%) serum and nasal swab samples were positive when tested using a bovine viral diarrhea virus (BVDV) antigen-capture ELISA kit. Two non-cytopathic strains of BVDV were isolated and named QL1903 and GA190608, respectively. The nucleotide sequences of the genomes of the two isolates were 89.52% identical. Phylogenetic analysis based on the 5'-UTR sequence revealed that the BVDV isolate QL1903 belonged to BVDV subtype 1b, whereas isolate GA190608 clustered with strains HN1814, EN-19, and BJ09_26 in a separate branch, which has tentatively been classified as a new genetic subtype, "1v".

**Supplementary Information:**

The online version contains supplementary material available at 10.1007/s00705-021-04990-7.

Bovine viral diarrhea virus (BVDV) is an immunosuppressive virus that is widely distributed throughout the world and poses an economic risk to the cattle industry [1]. It is responsible for a wide spectrum of clinical syndromes in cattle, including respiratory disease, reproductive dysfunction, immunosuppression, persistent infection, and mucosal disease [2]. BVDV, border disease virus (BDV) and classical swine fever virus (CSFV) belong to the genus *Pestivirus* within the family *Flaviviridae* [3]. Based on antigenic and genetic differences, two genotypes of BVDV have been identified: BVDV-1 and BVDV-2. Another group, referred to as “BVDV-3” or “atypical pestiviruses”, whose members are quite different from the above two genotypes, includes the strains Th/04_KhonKaen, D32/00-‘HoBi’, and others [4–6]. In addition, based on their ability to cause a cytopathic effect, BVDV-1, BVDV-2, and BVDV-3 isolates are categorized as either cytopathogenic (CP) or noncytopathogenic (NCP) [7, 8]. So far, 21 subgenotypes (BVDV-1a to BVDV-1u) have been reported worldwide [9, 10]. The BVDV genome is about 12.3 kb in length and comprises a 5' untranslated region (UTR), a 3'-UTR, and one open reading frame [11]. Due to the highly conserved nature of the 5′-UTR, the sequence of this region is widely used to determine the species and genotype of new pestivirus strains [12].

In March and June of 2019, calves on two cattle farms in Qionglai and Guang'an, Sichuan Province, developed clinical signs including diarrhea, anorexia, blood and intestinal mucosa in feces, and emaciation. Their condition did not improve significantly after antibiotic treatment, and they were clinically diagnosed as having a suspected BVDV infection. In order to determine the cause of diarrhea, 20 samples (10 serum samples and 10 nasal swabs) were collected from sick calves. The clinical manifestations of severe diarrhea in sick calves are shown in Fig. S1. All of the samples were examined for the presence of the viral E^rns^ glycoprotein using a BVDV Antigen Test Kit (IDEXX Laboratories), and two of the 20 samples were confirmed to be positive. The two BVDV-positive samples were processed for analysis by RT-PCR. Viral RNA was isolated using an EasyPure Viral DNA/RNA Kit (TransGen Biotech, China). *Trans Taq* HiFi PCR SuperMix (TransGen Biotech, China) and the primers 324 and 326 [13] were used for 5′-UTR amplification. This resulted in bright positive bands migrating above a 250-bp marker band (Fig. S2). A 288-bp region corresponding to the 5-UTR was sequenced by Sangon Biotech (Shanghai).

In order to isolate the virus and identify its biotype, the BVDV-positive samples were inoculated onto a monolayer of Madin-Darby bovine kidney (MDBK) cells in DMEM (Gibco, Life Technologies, USA) containing fetal bovine serum (FBS; Hyclone, Life Technologies USA) without bovine viral diarrhea virus antibodies or antigens. Cells were cultured at 37℃ with 5% CO_2_, and morphological changes in the cells were monitored each day. After one week of incubation, the lysates of the third-generation cells were collected to detect BVDV by RT-PCR. Two isolates (QL1903 and GA190608) were obtained that could be passaged stably in MDBK cells. QL1903 was isolated from a nasal swab sample from a calf in Qionglai. GA190608 was isolated from a serum sample from a calf in Guang'an. No cytopathic effect was observed in MDBK cells after inoculation with QL1903 or GA190608, suggesting that these two BVDV isolates belong to the NCP biotype. An indirect immunofluorescence assay performed as described previously [14] showed green staining in the cytoplasm of cells infected with these isolates (Fig. 1).

**Fig. 1:**
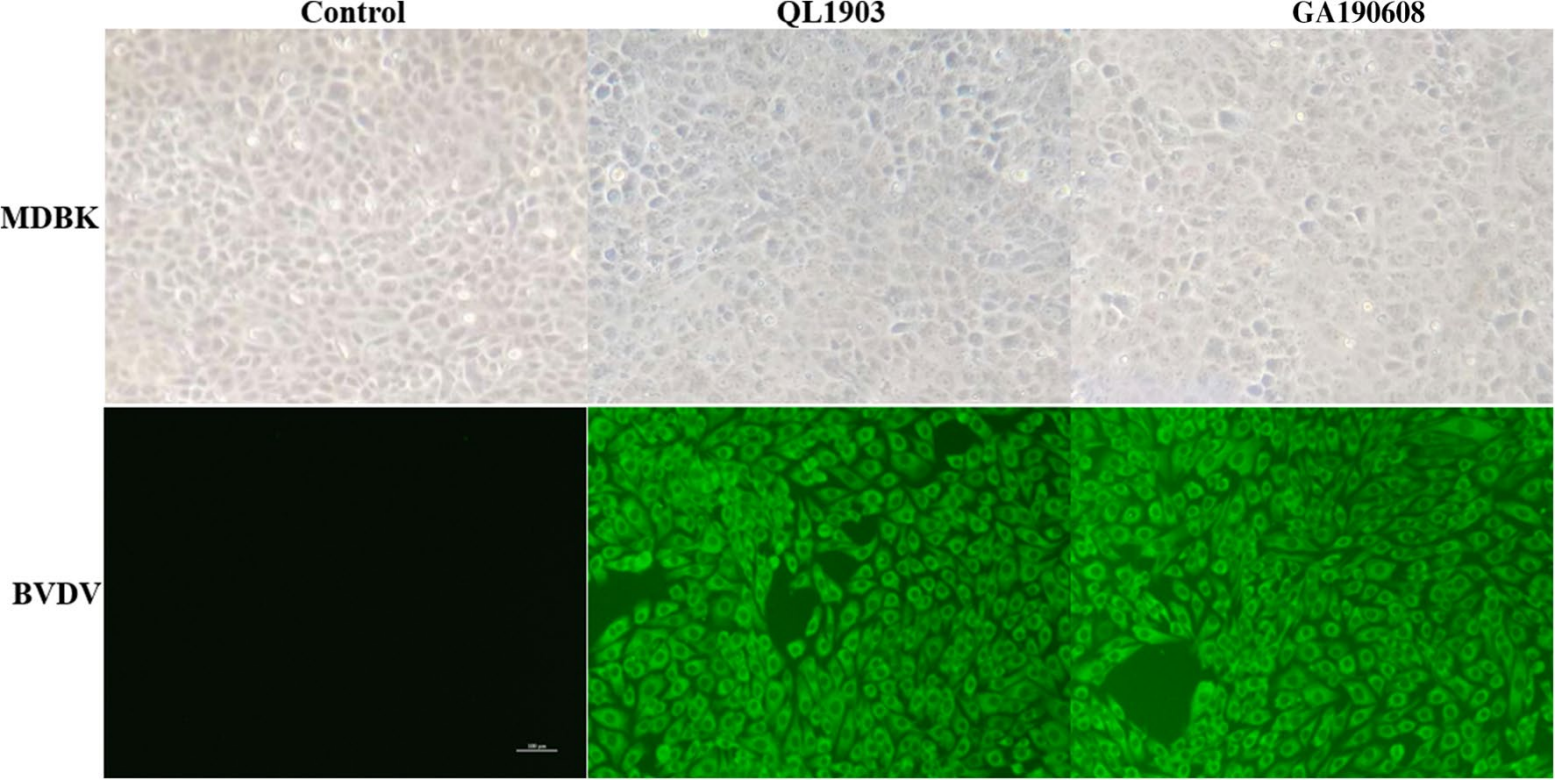
Immunofluorescence staining of BVDV in MDBK cells. The control group was not inoculated with BVDV-positive samples. The QL1903 group was inoculated with BVDV QL1903 samples. The GA190608 group was inoculated with BVDV GA190608 samples. BVDV antigens in the cytoplasm were stained green. Scale bar, 100 μm

To investigate the evolutionary relationship of the new isolates to BVDV isolates, phylogenetic analysis was performed based on 5′-UTR sequences available in the GenBank database. Nucleotide sequence comparisons were performed using NCBI BLAST online. Alignment and manual editing were performed using Molecular Evolutionary Genetics Analysis (MEGA) software, version 7. All of the sequences were aligned with BVDV reference strains retrieved from GenBank, including BVDV-1, BVDV-2, and BVDV-3, as well as CSFV and BDV. Phylogenetic trees were constructed using MEGA software as described previously [15]. The 5′-UTR sequence of the QL1903, and GA190608 isolates have been deposited in the GenBank database under accession numbers MN849041 and MT933204.

Phylogenetic analysis showed that the isolates QL1903 and GA190608 clustered with BVDV-1isolates (Fig. 2A). The nucleotide sequences of the two isolates were 89.52% identical to each other. The QL1903 isolate shared 94.72%-98.78% nucleotide sequence identity with the other BVDV-1 strains. QL1903 clustered with strains CC13B and HJ-1 in a branch (Fig. 2B) representing subgenotype 1b. QL1903 shared 94.09% and 95.93% nucleotide sequence identity with strains CC13B and HJ-1, respectively. QL1903 shared the highest nucleotide sequence identity (95.93%) with strain HJ-1 (KU756226), which was isolated in Heilongjiang Province in 2010. In the past years, BVDV subtype 1b has been detected frequently in Chinese bovine and porcine species [16, 17]. GA190608 shared 89.52%-98.37% nucleotide sequence identity with the other BVDV-1 strains and clustered with HN1814, EN-19, and BJ09_26 in a branch of the phylogenetic tree (Fig. 2B) that has not been assigned a genotype. GA190608 shared 98.67%, 98.76%, and 99.56% nucleotide sequence identity with HN1814, EN-19, and BJ09_26, respectively. GA190608 shared the highest nucleotide sequence identity (99.56%) with strain BJ09_26. According to data from GenBank, strain HN1814 was isolated from cattle in Henan Province in January 2020, EN-19 strain was isolated from ear notch samples from dairy cattle in China in 2017, and strain BJ09_26 (HQ116551) was isolated in Beijing in 2009. Since these three BVDV strains have not been assigned a subgenotype, we tentatively labelled the branch containing GA190608, HN1814, EN-19, and BJ09_26 as "BVDV-1v". Although these strains have not been assigned a genotype designation, they have been circulating recently in different areas of China. This may be related to the rapid development of the cattle industry and the transport of cattle from various places. This study is the first to report the emergence of the new subgenotype BVDV-1v, which can enrich the BVDV strains database and provide early warning to cattle disease control agencies.

**Fig. 2:**
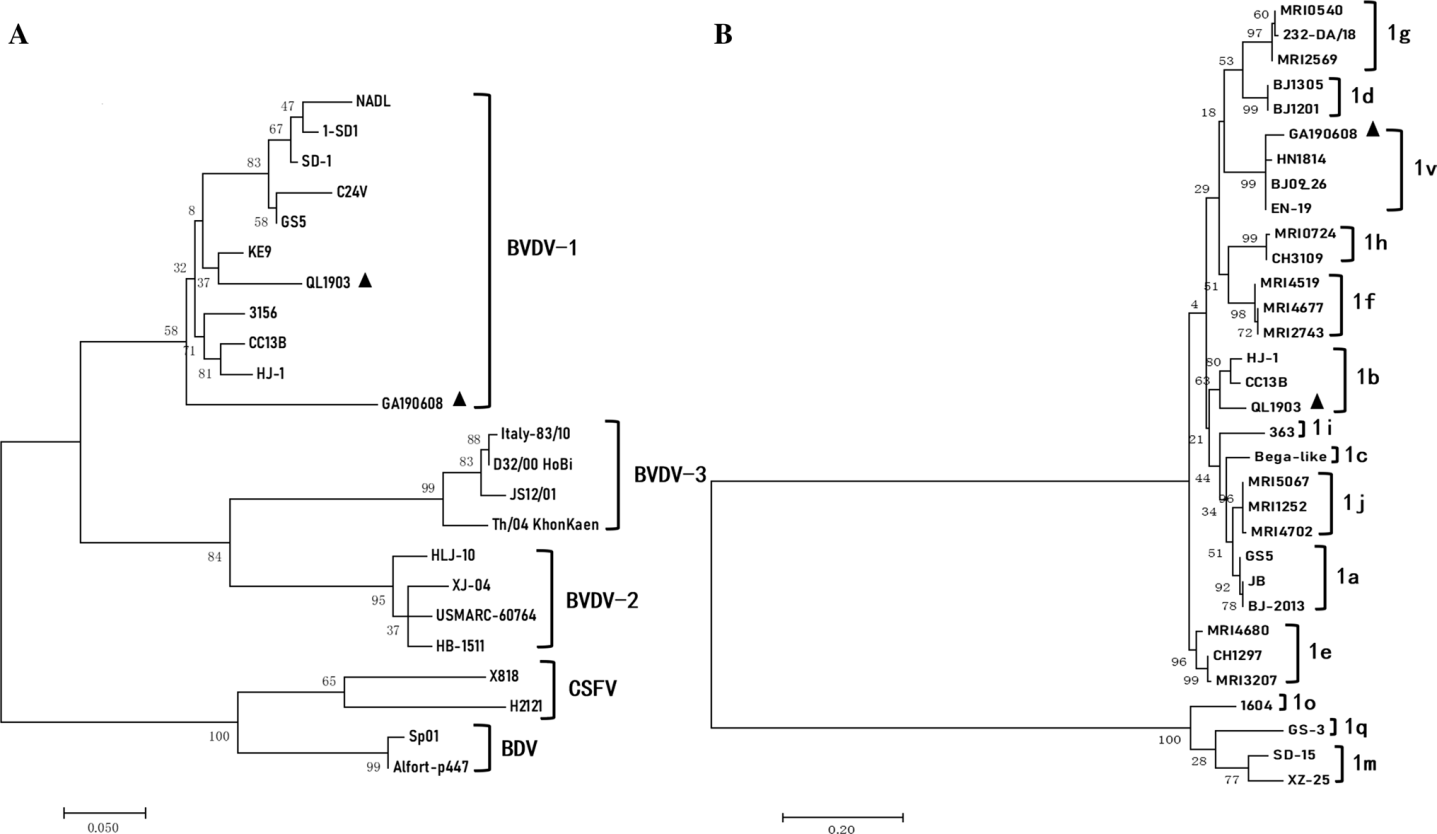
Phylogenetic tree constructed by the neighbor-joining method based on partial nucleotide sequences of the BVDV 5′-UTR. The QL1903 and GA190608 isolates are indicated by a black triangle. Another 51 sequences from pestiviruses reference strains (BVDV-1, BVDV-1, BVDV-3, CSFV, BDV) were retrieved from the GenBank database.

## Supplementary Information

Below is the link to the electronic supplementary material.

Fig. S1. The clinical symptoms of the diseased cattle (A) A sick calf discharged yellow watery loose feces in cattle farms of Qionglai; (B) A sick calf discharged black loose feces in cattle farms of Guang'an. (TIF 1328 KB)

Fig. S2. BVDV clinical isolates RT-PCR gel electrophoresis map (A) M: Marker, 1: negative control, Q1-Q3: 5'-UTR of QL1903; (B) M: Maker, 1: negative control, G1-G4: 5'-UTR of GA190608. (TIF 3395 KB)

Supplementary file3 (TXT 1 KB)

Supplementary file4 (TXT 1 KB)
