# Supplementary figures and images for: Identification and genotyping of a new subtype of bovine viral diarrhea virus 1 isolated from cattle with diarrhea
Source: Arch Virol. 2021 Feb 13;166(4):1259–62. doi: 10.1007/s00705-021-04990-7 (PMC7952330; doi:10.1007/s00705-021-04990-7)

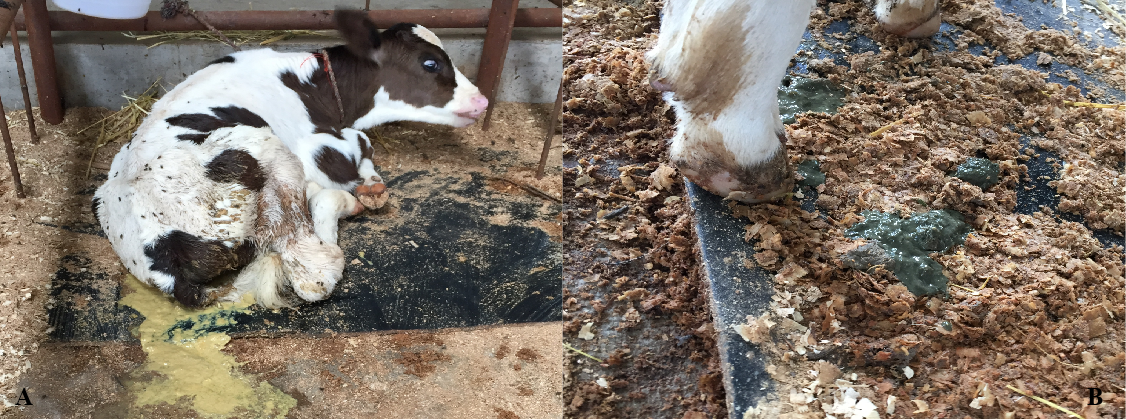

Supplement: Supplementary file 1 — Fig. S1. The clinical symptoms of the diseased cattle (A) A sick calf discharged yellow watery loose feces in cattle farms of Qionglai; (B) A sick calf discharged black loose feces in cattle farms of Guang'an. (TIF 1328 KB) [file 705_2021_4990_MOESM1_ESM.tif]

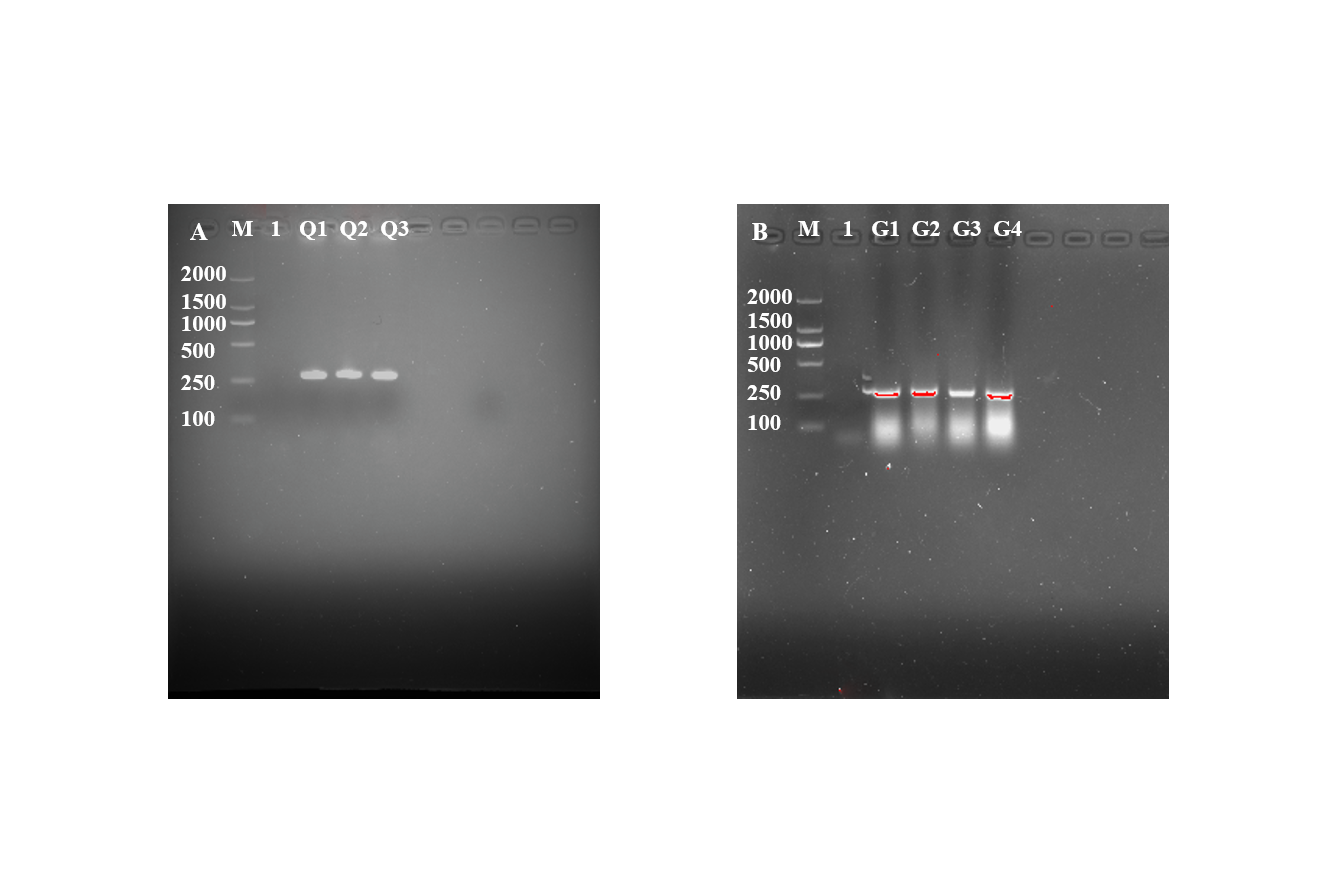

Supplement: Supplementary file 2 — Fig. S2. BVDV clinical isolates RT-PCR gel electrophoresis map (A) M: Marker, 1: negative control, Q1-Q3: 5'-UTR of QL1903; (B) M: Maker, 1: negative control, G1-G4: 5'-UTR of GA190608. (TIF 3395 KB) [file 705_2021_4990_MOESM2_ESM.tif]
